# Trait Variation in Moths Mirrors Small-Scaled Ecological Gradients in A Tropical Forest Landscape

**DOI:** 10.3390/insects11090612

**Published:** 2020-09-08

**Authors:** Dominik Rabl, Aura M. Alonso-Rodríguez, Gunnar Brehm, Konrad Fiedler

**Affiliations:** 1 Department of Botany & Biodiversity Research, University of Vienna, Rennweg 14, A-1030 Vienna, Austria; drabl@gmx.net; 2 Department of Field Station Fabrikschleichach, Biocenter, University of Würzburg, D-96181 Rauhenebrach, Germany; 3 The Gund Institute for Environment & Rubenstein School of Environment and Natural Resources, University of Vermont, Burlington, VT 05405, USA; aurapr15@gmail.com; 4 Institut für Zoologie und Evolutionsforschung, Phyletisches Museum, D-07743 Jena, Germany; gunnar.brehm@uni-jena.de

**Keywords:** Costa Rica, body size, mimicry rings, aposematism, oil palm plantations, lowland rainforest

## Abstract

**Simple Summary:**

Tropical rainforests are still lost at alarming rates due to timber extraction or conversion into plantations. While losses of species diversity are well documented, less is known about how the functional integrity of insect communities changes with such interventions. Using light-trap samples taken from species-rich moth assemblages in one region in SW Costa Rica, we asked whether the body size of moths and the contribution of warningly colored species change from old-growth forest across disturbed forest toward oil palm plantations. Across three topographic types of old-growth forest, differences were small. Moth species occurring in plantations were substantially smaller than their relatives thriving in natural forest. Similarly, the incidence of warning coloration dropped massively in plantations. Two different types of mimicry (moths imitating wasps or poisonous beetles, respectively) showed their own patterns of variation across ecosystems, yet both color types were very rare in plantations. These results confirm that not only insect species diversity becomes greatly diminished when tropical forests are destroyed: the functional composition and integrity of the insect fauna that remains in plantations is eroding as well.

**Abstract:**

Along environmental gradients, communities are expected to be filtered from the regional species pool by physical constraints, resource availability, and biotic interactions. This should be reflected in species trait composition. Using data on species-rich moth assemblages sampled by light traps in a lowland rainforest landscape in Costa Rica, we show that moths in two unrelated clades (Erebidae-Arctiinae; Geometridae) are much smaller-sized in oil palm plantations than in nearby old-growth forest, with intermediate values at disturbed forest sites. In old-growth forest, Arctiinae predominantly show aposematic coloration as a means of anti-predator defense, whereas this trait is much reduced in the prevalence in plantations. Similarly, participation in Müllerian mimicry rings with Hymenoptera and Lycidae beetles, respectively, is rare in plantations. Across three topographic types of old-growth forests, community-weighted means of moth traits showed little variation, but in creek forest, both types of mimicry were surprisingly rare. Our results emphasize that despite their mobility, moth assemblages are strongly shaped by local environmental conditions through the interplay of bottom–up and top–down processes. Assemblages in oil palm plantations are highly degraded not only in their biodiversity, but also in terms of trait expression.

## 1. Introduction

The concept of habitat filtering indicates that at any site in a landscape, organisms from the regional species pool will be the only ones able to build up a local community that withstands the environmental conditions prevailing there [1]. As a corollary, communities are expected to be assembled non-randomly in terms of traits that facilitate the establishment or survival of organisms in relation to abiotic or biotic factors that act as local ‘filters’. Hence, the study of species traits and their distribution across environmental gradients provides an opportunity to assess whether particular traits can explain how organisms surpass certain filters. Strong non-random patterns in the prevalence of traits can be viewed as an indicator of processes that act during community assembly.

Insect assemblages in tropical rainforests are usually highly species-rich, which poses a challenge for biodiversity studies. Some moth clades that are taxonomically better known provide a rather rare opportunity where species identifications are feasible even in speciose faunas, allowing us to pursue biodiversity studies along tropical environmental gradients with high taxonomic resolution [2,3,4]. A plethora of such studies during the past 2–3 decades have demonstrated that the species composition of these insects usually mirrors environmental gradients at surprisingly high spatial resolution. For example, in two earlier studies conducted in a perhumid tropical landscape in SW Costa Rica [5,6], we have shown that species composition reveals very clear patterns with regard to gradients in terms of land-use intensity and local topography. However, the ecological interpretation of such patterns is often hampered by the fact that for most tropical insect species, even if their taxonomy is reasonably well resolved, we lack information on many relevant life-history traits that would be required for a deeper functional understanding.

We here use these same Costa Rican moth assemblage data in connection with two sets of eco-morphological traits that can be easily generated from voucher samples to further explore differentiation along ecological gradients. Such traits may be more relevant for inferences on the roles that complex tropical insect assemblages play in ecosystem functioning than mere taxonomic identities. Specifically, we address the body size and various aspects of color patterns related to interactions with natural enemies. Body size is an extremely important trait for practically all animals [7,8]. In particular, larger body size usually indicates that the individual organisms require more resources to grow and sustain themselves, and it often takes more time to reach maturity than in smaller-sized relatives [9,10]. On the other hand, warning coloration and mimicry are two widespread strategies, especially among tropical insects, for defense against visually hunting predators [11]. Therefore, we expected that the expression of both these traits would vary, in a non-random manner, across different habitat types within the same landscape.

We tested the following three hypotheses:

(a) Larger moth species are more prevalent in intact lowland rainforest, whereas smaller species predominate in plantations that experience frequent cycles of anthropogenic intervention.

(b) Aposematism (warning coloration) is likewise more strongly expressed in intact rainforest, with its dense network of biotic interactions and high predation pressure.

(c) The incidence of mimicry, as a special case of warning colouration, follows the same spatial pattern as aposematism in general.

## 2. Material and Methods

### 2.1. Study Sites

Our study took place in the Pacific lowlands of the Golfo Dulce region in southwestern Costa Rica, near the Tropical Field Station La Gamba (8°43′ N, 83°13′ W). The study landscape included the margin of Piedras Blancas National Park, which is one of the last larger tracts of Pacific lowland wet rainforest of Central America [12] and its immediate surroundings. The regional climate is tropical perhumid, with an annual mean temperature of 28 °C, while mean precipitation amounts to approximately 6000 mm. There is a drier period from January to March and a peak in precipitation from August to November [13]. More detailed information about geography, geology, and climate of the Golfo Dulce region is provided in the monograph by Weissenhofer et al. [14].

We aimed at representing two complementary habitat gradients that are prevalent within this tropical landscape: first, a gradient of land-use intensity from old-growth forest across forest margin sites and young (<15 years old) spontaneous forest regrowth to oil palm plantations. Substantial tracts of former farmland and pastures have in recent years been converted into plantations of this latter cash crop [15,16]. Second, we investigated topographic gradients inside remaining old-growth forest, from creek forest stands situated in the narrow valleys, across mid-slope forest, to ridge forest sites. Recent research has confirmed the serious effects of oil palm cultivation on biodiversity of the region [5,17,18,19]. At the same time, there is a substantial variation in ecosystem structure and biota along the topographic gradient inside old-growth forests, even though these sites only span a narrow elevational range of about 100–280 m asl [6,20,21].

**Figure 1:**
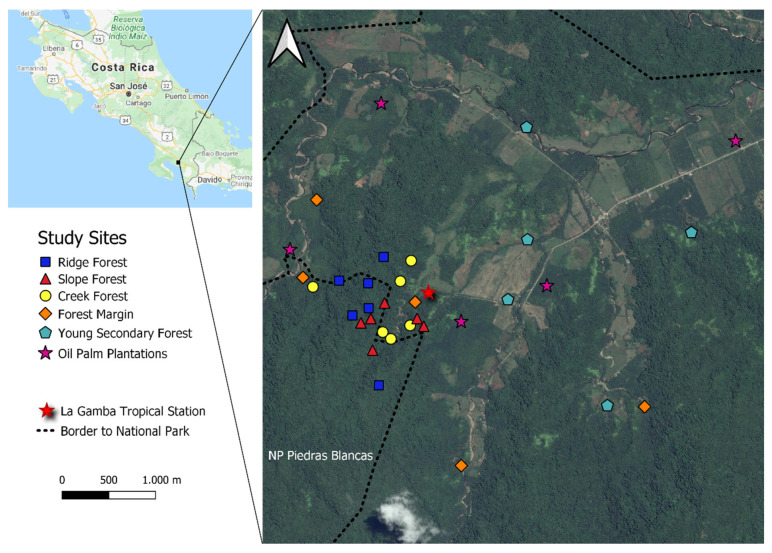
Schematic map showing the location of 38 light-trapping sites in the vicinity of the La Gamba Field Station, SW Costa Rica (8°43′ N, 83°13′ W).

In the year 2013, we selected 20 sites (5 replicates in each habitat type) representing old-growth forest, forest margins (FM), young secondary forest (YSF), and oil palm plantations (OPP). Of the five old-growth forest sites, two were later classified as creek (CF) and slope forest (SF), while one represented ridge forests (RF). In the following year 2014, we selected 18 additional sites for light-trapping, with 6 replicates each in the three forest categories CF, SF and RF. The distribution of the 38 sampling sites in the landscape is shown in Figure 1. Further information on the sites and their moth assemblages can be found in [5,6].

### 2.2. Moth Sampling and Processing

We sampled moths from February to July 2013 and again from July to October 2014 during the rainy season, using portable automated light traps (ca. 100 cm high; funnel opening: 6 cm diameter; with bucket; killing agent: chloroform). Each light trap was equipped with an 8 W blacklight tube, operated with a 12 V battery as power supply, and started through a twilight switch. For a detailed description of this trap, type see [22].

We placed light traps in the understory at 1–1.5 m aboveground. We performed six (2013) or seven (2014) collecting runs at each site. Weak light sources as used in this study minimize the cross-attraction of moths from habitats distant to the light trap [23]. To reduce the negative effects of moonlight on moth catches, we restricted sampling to four days prior to and after new moon nights (e.g., [24]. We emptied traps early in the morning to prevent ants and other insects from clearing out the bucket.)

Immediately after capture, moth specimens were frozen and later mounted and identified to species level. For the present study, we concentrated on two focal groups (Erebidae-Arctiinae and Geometridae) for which taxonomic information is sufficient to achieve species identifications and which are numerous enough in light-trap samples from the region to produce statistically meaningful samples. Arctiinae (lichen and tiger moths) and Geometridae (looper moths) have successfully served as model organisms in a range of biodiversity studies in the Neotropics [25,26].

Species identification was primarily achieved by comparison with voucher specimens, including the National Biodiversity Institute (INBio) in San José (Costa Rica), and using photographs of type material held in various scientific collections (most importantly: Smithsonian National Museum of Natural History (NMNH), Washington, D.C. and Natural History Museum (NHMUK), London). Some further species identifications were achieved using various online resources (e.g., http://www.discoverlife.org/mp/20q?guide=Moth_Costa_Rica, http://janzen.sas.upenn.edu/Wadults/search.lasso) and by consultation with taxonomic specialists (see Acknowledgments).

### 2.3. Data Collection and Statistical Analysis

We produced scaled digital photographs of all Geometridae and Arctiinae species identified in our samples (camera type: Nikon D 700, image resolution: 4256 × 2832 pixels, focal distance: 105 mm). Using the ImageJ 1.48v software (National Institute of Mental Health, Bethesda, Maryland, MD, USA), we measured the average forewing length of one to three male specimens per species (from the wing insertion point in the metathorax to the apex). We focused on males, since these make up the majority of specimens in light-trap catches, including our own samples. Specimens for analysis were selected to be well developed and with little wing wear to allow for proper measurements. Note that—as is usual for tropical insect samples—the majority of species was represented only by a few individuals. In our case, 33.3% of Arctiinae species and 42.2% of Geometridae species occurred in three or fewer individuals in the combined sample, therefore precluding any meaningful consideration of possible infraspecific body size variation in these cases. In the Arctiinae, to better cover potential body size variation, we measured 10 specimens each of the commonest species—always males that were sufficiently well preserved, allowing for precise wing measurements to be taken. Note that in these 20 species, body size variation was small (coefficient of variation between 1.77% and 7.21%, on average 4.21%). Therefore, we consider the selection of specimens used for the quantification of body size patterns at the community level sufficiently representative. We also determined wing width, thorax length and width, total body length, and various other metrics that could be derived from these measurements. Since these additional metrics were all highly correlated with forewing length, we hereafter only present results for this latter metric of body size.

**Figure 2:**
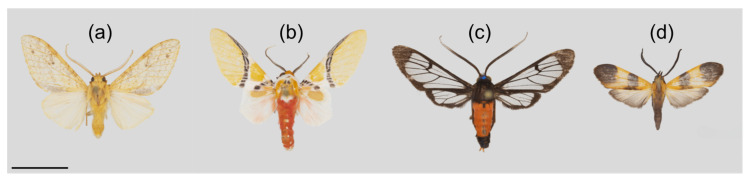
Exemplar species of Arctiinae scored as (**a**) cryptic, (**b**) aposematic, (**c**) Hymenopteran mimic, and (**d**) Lycidae beetle mimic. Species on display are (from left to right): *Lophocampa debilis* Schaus; *Idalus critheis* Druce; *Cosmosoma hector* Staudinger; *Correbidia* sp. nr. *terminalis* Walker. Scale bar: 1 cm.

In addition to body size, for all representatives of the Arctiinae we also scored the occurrence of aposematic coloration and their putative participation in mimicry rings (as binary variables). These color pattern traits were practically absent from the Geometridae species encountered in our samples. We scored Arctiinae moths as aposematically colored if they showed bright red or yellow markings on their wings or abdomen. If their wings had substantial transparent parts without scale cover, we scored them as hymenopteran mimics. Finally, they were scored as mimics of the beetle family Lycidae [27,28] if they resembled these insects. This trait is largely confined to the genera *Correbia* and *Correbidia* in our Arctiinae moth samples, with a few additional species in the genera *Dycladia*, *Lycomorphodes* and *Talara*. Exemplar species to represent these four categories are depicted in Figure 2. We did not observe in our samples any Arctiinae species where the sexes differed in regard to aposematism or inclusion in putative mimicry rings. Rather, without exception, all specimens available within a given species could be uniformly assigned to one of these color pattern categories. A full list of wing lengths and all scorings of Arctiinae moth species according to their aposematism and mimicry status is provided in the supplementary material to this article (Table S1), as are the measured forewing lengths of all Geometridae species (Table S2). We did not consider white species as being aposematic, because evidence is lacking that white lepidopteran species are generally less palatable for predators than camouflaged species [29].

Using these morphological data, we calculated the community weighted means (CWM) of forewing length, of the incidence of aposematism, and of the incidence of hymenopteran (and lycid) mimicry, separately for the moth assemblages of each light-trap site. Then, we explored, using Kruskal–Wallis ANOVA by ranks, whether the CWMs of the eco-morphological traits differed significantly between the moth assemblages from the various habitat types. All statistical analyses were conducted with the software PAST 4.03 [30].

## 3. Results

### 3.1. Body Size

**Figure 3:**
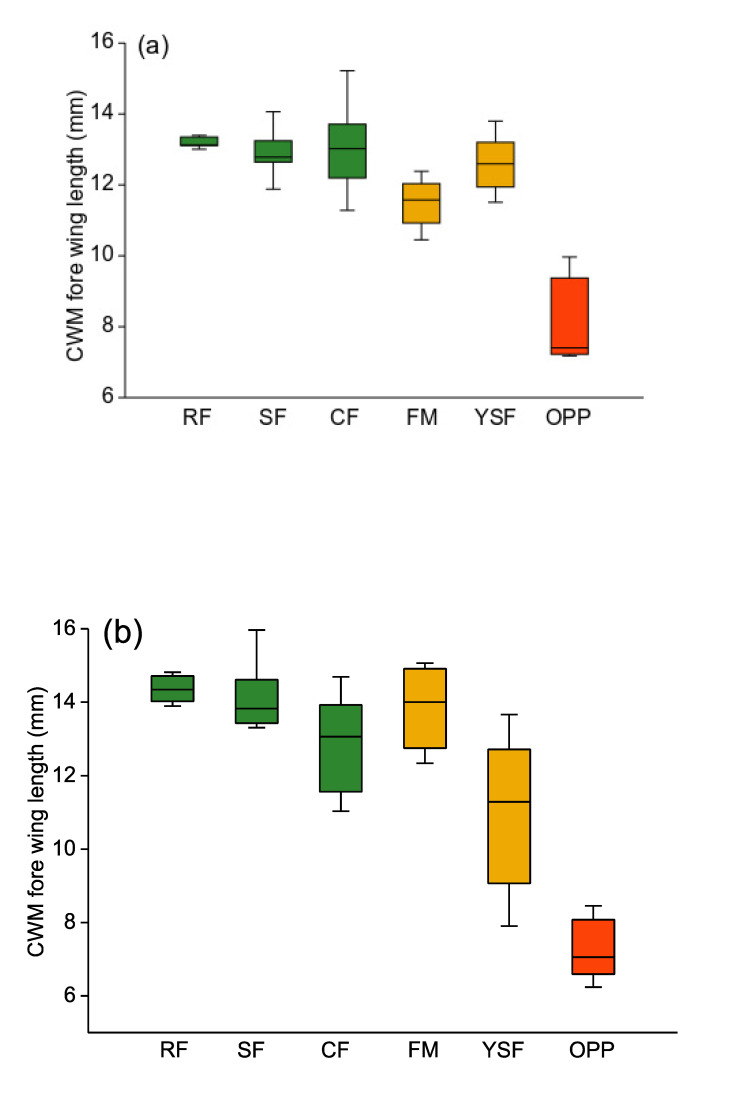
Box plots of community weighted means (CWM) of moth forewing length for Arctiinae (**a**) and Geometridae (**b**). Green: old-growth forest; orange: disturbed forest sites; red: oil palm plantations. RF—ridge forest; SF—slope forest; CF—creek forest; FM—forest margin; YSF—young secondary forest; OPP—oil palm plantations. Box: upper and lower quartile; horizontal line in box: median; whiskers: highest and lowest value.

Altogether, we observed 5570 individuals (171 species) of Arctiinae and 3846 individuals (258 species) of Geometridae. Variation in forewing length across species was substantial but quite similar in extent between the two focal moth clades: from 4.7 (*Prepiella* sp.) to 30.6 mm (*Ammalo* nr. *helops*) in Arctiinae, and from 3.8 mm (*Tricentrogyna* sp 03) to 36.2 mm (*Epimecis patronaria*) in Geometridae. CWMs of forewing length varied widely across the habitats under study. Arctiinae moth assemblages comprised species of similar, and rather large, average size in all three topographic types of old-growth forest (Figure 3a). At disturbed forest sites, such as forest margins and young regenerating forests, these assemblages consisted of slightly smaller moths. Finally, in oil palm plantations, CWMs of Arctiinae moth forewing length were by far the smallest (Kruskal–Wallis test: H_5df_ = 21.7, *p* = 0.0006).

Among Geometridae, the pattern of CWMs of forewing length across habitats was essentially similar (H_5df_ = 23.34, *p* = 0.0003; Figure 3b). Geometrids in oil palm plantations were again on average far smaller than in old-growth forest. Those at forest margin sites were similar in size to old-growth forest communities, whereas in young secondary forests, geometrid average body size was a bit lower and highly variable between individual sites. CWMs of forewing length of Arctiinae and Geometridae moths were highly significantly correlated across the 38 study sites (Pearson’s r = 0.7082, *p* < 0.0001).

### 3.2. Incidence of Aposematism in Arctiinae

**Figure 4:**
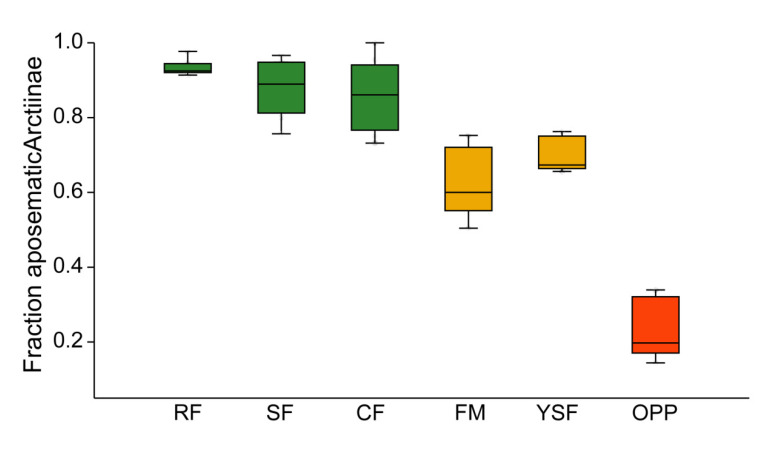
Box plots of community weighted means of aposematic coloration among Arctiinae assemblages. Green: old-growth forest; orange: disturbed forest sites; red: oil palm plantations. RF—ridge forest; SF—slope forest; CF—creek forest; FM—forest margin; YSF—young secondary forest; OPP—oil palm plantations. Box: upper and lower quartile; horizontal line in box: median; whiskers: highest and lowest value.

In all three topographic variants of old-growth forest, the vast majority of tiger and lichen moths were aposematically colored (Figure 4). In the two types of anthropogenically disturbed forest habitats, the incidence of aposematism was lower but still high, usually >50%. In contrast, aposematic coloration in oil palm plantations pertained only to a minority of Arctiinae. These differences between habitat types were highly significant (H_5df_ = 27.91, *p* < 0.0001).

### 3.3. Incidence of Mimicry Among Arctiinae

**Figure 5:**
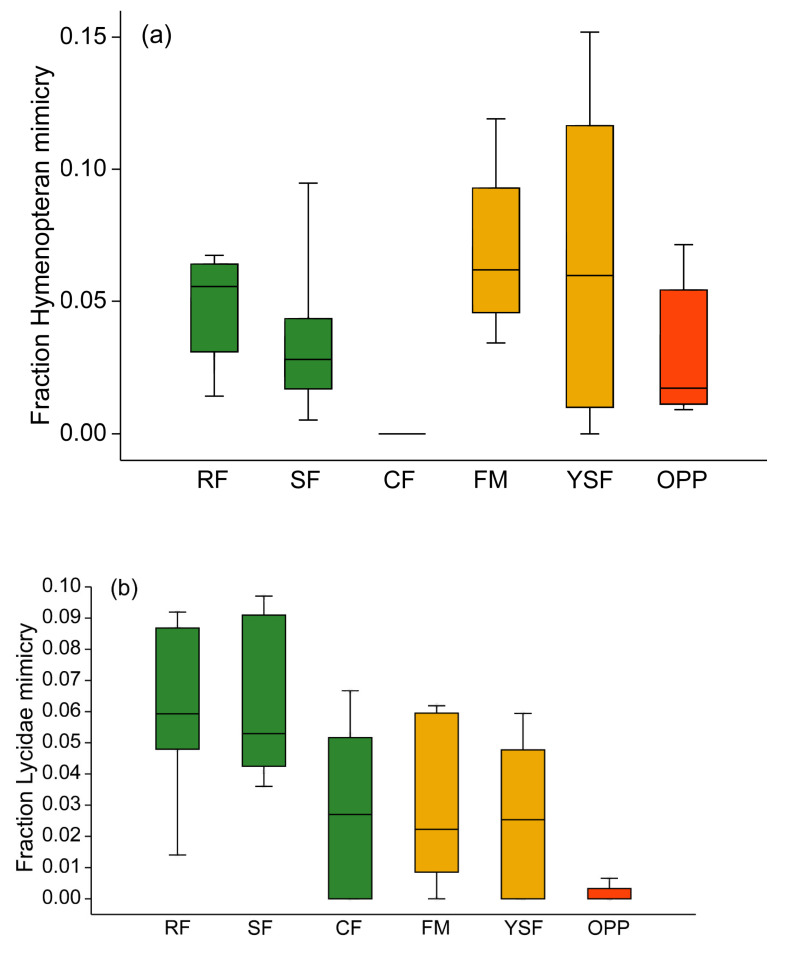
Box plots of community weighted means of hymenopteran mimicry (**a**) and Lycidae beetle mimicry (**b**) among Arctiinae assemblages. Green: old-growth forest; orange: disturbed forest sites; red: oil palm plantations. RF—ridge forest; SF—slope forest; CF—creek forest; FM—forest margin; YSF—young secondary forest; OPP—oil palm plantations. Box: upper and lower quartile; horizontal line in box: median; whiskers: highest and lowest value.

Arctiinae showing hymenopteran mimicry (i.e., with transparent non-scaled wing fractions) were unequally distributed across the six types of forest habitats (H_5df_ = 20.73, *p* = 0.0009). Within old-growth forest, these moths were most prevalent at ridge sites, slightly less common at slope sites, but completely absent at creek forest (Figure 5a). At disturbed forest sites, hymenopteran mimicry was not less common than in old-growth forest, but in oil palm plantations, few tiger moths mimicked bees or wasps. Members of the mimicry ring involving Lycidae beetles were rather rare overall, but they showed a similar unequal distribution across forest types as the hymenopteran mimics (H_5df_ = 16.23, *p* = 0.0062; Figure 5b). They were less common in creek forest than in the two other topographic types of old-growth forest, and in oil palm plantations, moths of the genera *Correbia* and *Correbidia* were rarely encountered at all. Across the 38 study sites, the relative prevalence of hymenopteran mimicry amongst Arctiinae moths was not related to the incidence of lycid beetle mimicry (Pearson’s r = 0.1661, *p* = 0.319).

## 4. Discussion

Oftentimes, variation in eco-morphological traits reflects ecosystem processes and thereby reveals stressors or constraints acting upon community assembly and ecosystem functioning [31]. Yet only a few studies have thus far examined biometrical differences of moths along environmental gradients in tropical rainforests (e.g., vertical stratification [32]; elevational gradients [22,26,33]; primary forest and margins [25]). Even fewer studies have attempted to trace such patterns along tropical land-use gradients (for Amazonian dung beetles [34]). We have shown above that the body size of Neotropical moths and color patterns linked to anti-predator defense functions differ strongly between natural old-growth forest and more degraded forest habitats. These patterns are suggestive of a steep decline in the functioning of ecological networks into which moth assemblages are embedded, particularly in intensively managed oil palm plantations.

### 4.1. Body Size

Average wing length within moth assemblages was very similar in all three types of old-growth forest habitats, but it was substantially smaller in oil palm plantations in both arctiines and geometrids. This pattern was largely the same for these two distantly related moth clades that differ in a variety of their life-history traits. Possible reasons for the disproportionate selective loss of larger moths in oil palm plantations are (1) reduced food availability, (2) longer generation times of larger moths, and (3) their higher predation risk due to a lack of shelters to hide during the daytime.

(1) Herbicide-treated oil palm monocultures are lacking understory vegetation diversity and structure [5], which are both features characterizing natural tropical forest habitats. Due to the absence of any substantial understory vegetation, which consists of different sized trees, bushes, lianas and herbs in natural forests, food resource availability for developmental stages of moth species is probably severely reduced. Larger caterpillars require more food and cannot easily develop on the sparse herbal vegetation or on lichens, which are the only potential food resources for Lepidopteran larvae to remain in oil palm plantations, apart from the planted palm trees. This appears to be true even though larger species in Geometridae such as *Oxydia* tend to be polyphagous, whereas smaller species are often specialized feeders in their larval stages. As an example, species of the geometrid genus *Eois* are usually small in size and are specialist feeders of Piperaceae plants [35]. While *Eois* moths made up a sizeable fraction of our light trap samples in old-growth forests, only two *Eois* species ever appeared in oil palm plantations.

(2) The generation times of larger moth species are usually longer than for small-sized species [36]. On the one hand, the longer caterpillar development takes, the higher is their risk to fall victim to one of the many management rotations performed every year inside the plantations [37]. On the other hand, this implies that populations of large species recover too slowly after frequent applications of herbicides and insecticides.

(3) Although species richness of insectivorous predators declines dramatically in oil palm plantations (amphibians and reptiles [17]; bats [18]; birds [38]), disturbance-tolerant generalist vertebrate species still exist there and effectively reduce the populations of herbivorous insects [39]). The impoverished vegetation structure of oil palm monocultures does not provide the necessary hiding places (e.g., leaf litter, woody debris, understory plants) for caterpillars, pupae, and adult moths (especially larger species), making them more apparent to visually hunting predators such as reptiles and birds. For many insectivorous bats, maneuvering and hunting is easier in the more open vegetation structure of oil palm plantations or in disturbed open forest habitats [18,40], which may also increase the relative impact of these predators on populations of large-sized moths. On the other hand, ants also severely decline in oil palm plantations, and therefore, their role as predators of moth larvae is reduced, relative to old-growth forest (for the Golfo Dulce region [19]).

The observed decline in the body size of Neotropical moths relative to old-growth forest may have profound implications for the ecological functioning in disturbed forest habitats and even more so in oil palm plantations. With the degradation of moth faunas in diversity, abundance, and body size, more specialist insectivorous predators lose an important food source, so that food webs in highly disturbed tropical forest systems are seriously modified [41]. In contrast, although understory density within the closed old-growth forest varies between topographical types [6,14], differences between these forest types were too subtle and the habitat fidelity of most moth species was too limited to measurably influence community-wide moth size within intact rainforests at this small spatial scale.

### 4.2. Aposematism

Aposematic Arctiinae moths dominated the respective assemblages in all three topographic variants of old-growth forest, but they featured less prominently in anthropogenically disturbed forest habitats and almost disappeared in oil palm plantations. We here suggest a potential explanation for this pattern that relates to the widespread occurrence of acquired chemical defense in this moth clade [42,43]. Many species of Arctiinae moths sequester secondary plant metabolites from their toxic larval host plants. The sequestration of plant toxins may also occur through adult moths (so-called pharmacophagy), in order to obtain protection against predators as well as to harvest basic components required for sex pheromone synthesis [44]. These complex insect–plant interactions can only function if the appropriate plant resources are available in sufficient supply. Therefore, it seems likely that Arctiinae moths are not able to sequester the plant toxins they require to become unpalatable in structurally impoverished habitats such as oil palm plantations.

The concentration of sequestered secondary plant metabolites in the host plants where the larvae are feeding plays a crucial role for enhancing anti-predator defense and decides if and to what extent they gain unpalatability [45]. Although there is a trade-off between the production and maintenance of coloration and chemical defense [46], wing color patterns of arctiines are genetically determined [47], and they are usually diagnostic at the species level (i.e., wing colors are not plastic between different local environments). Therefore, individuals of aposematic colored arctiine species that are unable to acquire chemical defenses become an “easy prey” for predators. This disadvantage is likely exacerbated in the open vegetation structure of degraded forest habitats and oil palm plantations, which provide higher light availability for visually hunting predators [48]. Visually hunting predators are the major selective factor for the evolution of aposematic coloration in Lepidoptera, resulting in a great diversity of aposematic species within complex structured tropical rainforests, where warning colors ensure effective protection for unpalatable species and their Batesian mimics [11,49,50]. Low vegetation diversity and structural complexity, and the concomitant loss of quantity and quality of food resources in highly degraded tropical forest systems, leads to a diametrically opposed situation where warning colors lose their ecological function and may even accelerate diversity loss through facilitated predation.

### 4.3. Hymenopteran and Lycid Mimicry Rings

Contrary to our expectations, the spatial pattern in the occurrence of aposematism did not closely match the pattern of involvement of these same moths in mimicry rings. In addition, the extent to which tiger moths of local assemblages were involved in hymenopteran mimicry rings was completely unrelated to the occurrence of lycid beetle mimicry. The prevalence of tiger moths showing hymenopteran mimicry was as high in disturbed forest habitats as in old-growth forests, but these tiger moths were barely present in oil palm plantations. In contrast, lycid mimics were slightly less prevalent at forest margins and in young secondary forests when compared to old-growth forests, and they were again nearly lacking in oil palm plantations. Remarkably, representatives of both mimicry rings were entirely absent or had a low prevalence in creek forests. However, these forests tend to show strong vertical stratification [51]. Therefore, moths participating in mimicry rings may primarily occur in the canopy and would have been missed by our understory-focused sampling. These non-random distributions of mimicry rings and their low match to overall aposematism suggest that beyond phylogenetic constraints, these two related functional traits may be driven by different mechanisms that require further study.

Lycid beetles and tiger moths showing lycid mimicry (mainly from the genera *Correbia* and *Correbidia*) are chemically well defended, forming a Müllerian mimicry ring where all members benefit from the unpalatability of the others [27,52]. Similar to other aposematically colored moths, lycid beetles and their mimics need a diversity of toxic plants to sequester secondary metabolites. Caterpillars in *Correbia* and *Correbidia* feed on *Ficus* and *Cecropia* trees, respectively (http://janzen.sas.upenn.edu/caterpillars/database.lasso). These two tree genera are represented by multiple species in all forest types around La Gamba. Yet, a lack of potential larval host plants might explain why they were almost completely absent in oil palm plantations. Overall, these lycid mimics were uncommon, accounting for less than 10% of tiger moth individuals.

In addition to having aposematic colors and sequestering toxic secondary compounds from plants, hymenopteran mimics imitate well-defended stinging wasps and bees [53]. This additional protection against visually hunting predators could be the reason why arctiines showing hymenopteran mimicry have experienced a less steep decline in the more open structure of disturbed forest habitats and in oil palm monocultures, where their putative models still thrive to some extent. The rarity of hymenopteran mimics in the understory of creek forests might again be explained by the vertical stratification of assemblages [51]. Another possible reason is that the activity of visually hunting predators is higher in the more open forest types and lower in the creeks with their particularly dense vegetation structure, but this hypothesis needs further testing.

## 5. Conclusions

Negative impacts on biodiversity due to the deforestation and intensification of land use in tropical regions are a well-observed phenomenon. Yet, eco-morphological features along anthropogenic disturbance gradients, which give deeper insights into ecosystem processes and functioning, have more rarely been evaluated. We investigated selected eco-morphological traits along a gradient of land conversion from old-growth tropical forest to intensively managed oil palm monocultures, demonstrating that forest degradation has a cascade of feedbacks concerning insect body size and traits related to predator avoidance. Our results suggest that the maintenance of a high vegetation complexity and diversity provided by natural tropical rain forests is indispensable to preserve the ecological functions and species diversity of tropical insects and should be considered highest priority for conservation plans.

## Supplementary Materials

The following are available online at https://www.mdpi.com/2075-4450/11/9/612/s1, Table S1: Measured fore wing length and scoring of aposematism and mimicry types of all Arctiinae species in the samples. Table S2: measured fore wing length of all Geometridae species in the samples.
