# Supplementary material for: Trait Variation in Moths Mirrors Small-Scaled Ecological Gradients in A Tropical Forest Landscape
Source: Insects. 2020 Sep 8;11(9):612. doi: 10.3390/insects11090612 (PMC7563231; doi:10.3390/insects11090612)
Supplement: Supplementary file 1 [file insects-11-00612-s001.zip › Table S1_revised.docx]

**Table S1:** Fore-wing length, status of aposematic coloration, and involvement in mimicry rings of 171 species of moths in the subfamily Erebidae-Arctiinae, sampled with light-traps along two environmental gradients in the region around La Gamba (SW Costa Rica).

| **Tribe** | **Species name** | **Fore-wing  length (mm)** | **Aposematic color present** | **Hymenopteran mimic** | **Lycidae beetle mimic** |
| --- | --- | --- | --- | --- | --- |
| Arctiini | *Abrochia moza* | 13.3 | 1 | 1 | 0 |
| Arctiini | *Aclytia albistriga* | 14.3 | 1 | 0 | 0 |
| Arctiini | *Aclytia gynamorpha* | 15.4 | 1 | 0 | 0 |
| Arctiini | *Aclytia punctata* | 13.6 | 1 | 0 | 0 |
| Arctiini | *Agaraea* nr. *minuta* | 7.9 | 0 | 0 | 0 |
| Arctiini | *Agaraea semivitrea* | 16.7 | 1 | 0 | 0 |
| Arctiini | *Amaxia apyga* | 15.6 | 0 | 0 | 0 |
| Arctiini | *Ammalo* nr. *helops* | 30.6 | 1 | 0 | 0 |
| Arctiini | *Amphelarctia priscilla* | 17.9 | 1 | 0 | 0 |
| Arctiini | *Baritius* nr. *eleuthera* | 13.8 | 1 | 0 | 0 |
| Arctiini | *Cercopimorpha sylva* | 14.3 | 1 | 0 | 0 |
| Arctiini | *Cissura plumbea* | 17.1 | 1 | 0 | 0 |
| Arctiini | *Correbia* nr. *fulvescens* | 15.9 | 1 | 0 | 1 |
| Arctiini | *Correbia* sp01 | 17.2 | 1 | 0 | 1 |
| Arctiini | *Correbia* sp02 nr. *undulata* | 18.1 | 1 | 0 | 1 |
| Arctiini | *Correbia* sp03 | 19.0 | 1 | 0 | 1 |
| Arctiini | *Correbia* sp04 | 17.3 | 1 | 0 | 1 |
| Arctiini | *Correbidia costinotata* | 14.7 | 1 | 0 | 1 |
| Arctiini | *Correbidia terminalis* | 11.4 | 1 | 0 | 1 |
| Arctiini | *Cosmosoma achemon* | 13.2 | 1 | 1 | 0 |
| Arctiini | *Cosmosoma angustimargo* | 15.5 | 1 | 1 | 0 |
| Arctiini | *Cosmosoma caecum* | 19.4 | 1 | 1 | 0 |
| Arctiini | *Cosmosoma hector* | 16.0 | 1 | 1 | 0 |
| Arctiini | *Cosmosoma* nr. *gemmata* | 16.7 | 1 | 1 | 0 |
| Arctiini | *Cosmosoma teuthras* | 19.8 | 1 | 1 | 0 |
| Arctiini | *Cosmosoma xanthostictum* | 13.1 | 1 | 1 | 0 |
| Arctiini | *Delphyre testacea* | 17.5 | 1 | 0 | 0 |
| Arctiini | *Dycladia vitrina* | 14.5 | 1 | 0 | 1 |
| Arctiini | *Dysschema jansonis* | 24.6 | 1 | 0 | 0 |
| Arctiini | *Dysschema panamensis* | 27.2 | 1 | 0 | 0 |
| Arctiini | *Elysius conspersus* | 26.7 | 1 | 0 | 0 |
| Arctiini | *Elysius discoplaga* | 19.8 | 1 | 0 | 0 |
| Arctiini | *Epanycles imperialis* | 13.1 | 1 | 0 | 0 |
| Arctiini | *Epidesma* nr. *ursula* | 18.5 | 1 | 0 | 0 |
| Arctiini | *Epidesma sixola* | 14.4 | 1 | 0 | 0 |
| Arctiini | *Epidesma* sp01 nr. *oceola* | 16.2 | 1 | 0 | 0 |
| Arctiini | *Epidesma* sp02 | 13.9 | 1 | 0 | 0 |
| Arctiini | *Episcepsis aelia* | 13.0 | 1 | 0 | 0 |
| Arctiini | *Episcepsis capysca* | 14.4 | 1 | 0 | 0 |
| Arctiini | *Episcepsis demonis* | 18.8 | 1 | 0 | 0 |
| Arctiini | *Episcepsis hypoleuca* | 15.6 | 1 | 0 | 0 |
| Arctiini | *Episcepsis thetis/inornata* | 15.3 | 1 | 0 | 0 |
| Arctiini | *Episcepsis xanthura* | 16.4 | 1 | 0 | 0 |
| Arctiini | *Eucereon aeolum* | 13.0 | 1 | 0 | 0 |
| Arctiini | *Eucereon aoris* | 13.8 | 1 | 0 | 0 |
| Arctiini | *Eucereon atrigutta* | 13.5 | 1 | 0 | 0 |
| Arctiini | *Eucereon aurantiaca* | 16.3 | 1 | 0 | 0 |
| Arctiini | *Eucereon latisfasciata* | 17.2 | 1 | 0 | 0 |
| Arctiini | *Eucereon maia* | 13.4 | 1 | 0 | 0 |
| Arctiini | *Eucereon mitigatum* | 15.0 | 1 | 0 | 0 |
| Arctiini | *Eucereon* nr. *obscurata* | 11.9 | 1 | 0 | 0 |
| Arctiini | *Eucereon* nr. *varium* | 16.4 | 1 | 0 | 0 |
| Arctiini | *Eucereon pometina* | 12.6 | 0 | 0 | 0 |
| Arctiini | *Eucereon pseudarchias* | 20.0 | 1 | 0 | 0 |
| Arctiini | *Eucereon relegata* | 16.5 | 1 | 0 | 0 |
| Arctiini | *Eucereon rosinum* | 12.2 | 1 | 0 | 0 |
| Arctiini | *Eucereon* sp01 | 13.4 | 1 | 0 | 0 |
| Arctiini | *Eucereon* sp02 | 16.3 | 1 | 0 | 0 |
| Arctiini | *Eucereon tesselata* | 16.2 | 1 | 0 | 0 |
| Arctiini | *Eupseudosoma aberrans* | 19.2 | 1 | 0 | 0 |
| Arctiini | *Evius hippia* | 15.1 | 1 | 0 | 0 |
| Arctiini | *Gymnelia jansonis* | 14.7 | 1 | 1 | 0 |
| Arctiini | *Halysidota fumosa* | 23.6 | 1 | 0 | 0 |
| Arctiini | *Heliura rhodophila* | 13.1 | 1 | 0 | 0 |
| Arctiini | *Heliura* nr. *phaeosoma* | 13.2 | 1 | 0 | 0 |
| Arctiini | *Heliura thysbodes* | 15.3 | 1 | 0 | 0 |
| Arctiini | *Hyalurga sixola* | 23.0 | 1 | 0 | 0 |
| Arctiini | *Hyalurga sora* | 20.7 | 1 | 0 | 0 |
| Arctiini | *Hyalurga urioides* | 21.9 | 1 | 0 | 0 |
| Arctiini | *Hyperandra excavata* | 15.2 | 1 | 0 | 0 |
| Arctiini | *Hypercompe* sp | 23.0 | 1 | 0 | 0 |
| Arctiini | *Hypocrita arcaei* | 24.7 | 1 | 0 | 0 |
| Arctiini | *Ichoria* sp. | 16.1 | 1 | 1 | 0 |
| Arctiini | *Idalus crinis* | 11.8 | 1 | 0 | 0 |
| Arctiini | *Idalus critheis* | 14.1 | 1 | 0 | 0 |
| Arctiini | *Idalus tybris* | 18.8 | 1 | 0 | 0 |
| Arctiini | *Isanthrene crabroniformis* | 22.9 | 1 | 1 | 0 |
| Arctiini | *Isanthrene* nr. *azia* | 15.5 | 1 | 1 | 0 |
| Arctiini | *Isanthrene* nr. *fulvipicta* | 17.4 | 1 | 1 | 0 |
| Arctiini | *Kirrostola metaxantha* | 12.7 | 1 | 0 | 0 |
| Arctiini | *Leucanopsis cedon* | 21.0 | 0 | 0 | 0 |
| Arctiini | *Leucanopsis* nr. *polydonta* | 17.2 | 0 | 0 | 0 |
| Arctiini | *Lophocampa debilis* | 15.7 | 0 | 0 | 0 |
| Arctiini | *Lophocampa* nr. *maroniensis* | 15.2 | 0 | 0 | 0 |
| Arctiini | *Lophocampa* nr. *modesta* | 14.0 | 0 | 0 | 0 |
| Arctiini | *Lophocampa subannula* | 12.6 | 0 | 0 | 0 |
| Arctiini | *Loxophlebia flavipicta* | 10.3 | 1 | 1 | 0 |
| Arctiini | *Loxophlebia* nr. *flavipicta* | 8.7 | 1 | 1 | 0 |
| Arctiini | *Loxophlebia* sp. | 11.6 | 1 | 1 | 0 |
| Arctiini | *Lymire* nr. *fulvicollis* | 12.5 | 0 | 0 | 0 |
| Arctiini | *Macrocneme iole* | 16.4 | 1 | 1 | 0 |
| Arctiini | *Macrocneme lades* | 15.5 | 1 | 1 | 0 |
| Arctiini | *Macrocneme* sp. | 15.7 | 1 | 1 | 0 |
| Arctiini | *Melese laodamia* | 16.4 | 1 | 0 | 0 |
| Arctiini | *Melese* nr. *incertus* | 12.7 | 1 | 0 | 0 |
| Arctiini | *Melese sixola* | 10.9 | 1 | 0 | 0 |
| Arctiini | *Mydromera notochloris* | 17.5 | 1 | 1 | 0 |
| Arctiini | *Napata leucotelus* | 10.5 | 1 | 1 | 0 |
| Arctiini | *Notarctia proxima* | 18.7 | 1 | 0 | 0 |
| Arctiini | *Ochrodota pronapides* | 11.6 | 0 | 0 | 0 |
| Arctiini | *Ormetica ataenia* | 16.2 | 1 | 0 | 0 |
| Arctiini | *Ormetica guapisa* | 17.9 | 1 | 0 | 0 |
| Arctiini | *Ormetica sicilia* | 17.3 | 1 | 0 | 0 |
| Arctiini | *Pareuchaetes insulata* | 15.6 | 1 | 0 | 0 |
| Arctiini | *Pelochyta* nr. *cervina* | 16.9 | 1 | 0 | 0 |
| Arctiini | *Pelochyta* nr. *ruficollis* | 22.8 | 1 | 0 | 0 |
| Arctiini | *Phaeomolis vampa* | 15.5 | 1 | 0 | 0 |
| Arctiini | *Phoenicoprocta sanguineum* | 13.6 | 1 | 1 | 0 |
| Arctiini | *Poliopastea* sp. | 13.9 | 1 | 1 | 0 |
| Arctiini | *Pompiliodes* nr. *postica* | 10.2 | 0 | 1 | 0 |
| Arctiini | *Pseudomya melanthoides* | 8.0 | 1 | 1 | 0 |
| Arctiini | *Psoloptera basifulva* | 15.5 | 1 | 1 | 0 |
| Arctiini | *Ripha flammans* | 24.0 | 1 | 0 | 0 |
| Arctiini | *Robinsonia bartolana* | 19.4 | 1 | 0 | 0 |
| Arctiini | *Robinsonia* sp. | 16.0 | 0 | 0 | 0 |
| Arctiini | *Saurita* nr. *concisa* | 8.5 | 1 | 1 | 0 |
| Arctiini | *Saurita phoenicosticta* | 11.5 | 1 | 1 | 0 |
| Arctiini | *Saurita tipulina* | 8.6 | 0 | 1 | 0 |
| Arctiini | *Sutonocrea* nr. *lobifer* | 14.6 | 1 | 0 | 0 |
| Arctiini | *Symphlebia ipsea* | 17.7 | 1 | 0 | 0 |
| Arctiini | *Thysanoprymna haemorrhoides* | 15.4 | 1 | 0 | 0 |
| Arctiini | *Timalus caeruleus* | 21.5 | 1 | 1 | 0 |
| Arctiini | *Trichromia cardinalis* | 14.7 | 1 | 0 | 0 |
| Arctiini | *Trichromia lophosticta* | 13.7 | 1 | 0 | 0 |
| Arctiini | *Trichromia* nr. *flavimargo* | 12.7 | 1 | 0 | 0 |
| Arctiini | *Trichromia* nr. *flexuosa* | 11.4 | 1 | 0 | 0 |
| Arctiini | *Trichromia* nr. *granatina* | 11.6 | 1 | 0 | 0 |
| Arctiini | *Trichromia* nr. *peninsulata* | 10.3 | 1 | 0 | 0 |
| Arctiini | *Trichromia parnelli* | 10.9 | 1 | 0 | 0 |
| Arctiini | *Trichromia* sp. | 9.9 | 1 | 0 | 0 |
| Arctiini | *Trichromia tipolis* | 15.3 | 1 | 0 | 0 |
| Arctiini | *Trichura* sp. | 17.1 | 1 | 1 | 0 |
| Arctiini | *Tricypha imperialis* | 16.4 | 1 | 0 | 0 |
| Arctiini | *Uranophora walkeri* | 13.8 | 1 | 0 | 0 |
| Arctiini | *Virbia* nr. *mentiens* | 12.7 | 1 | 0 | 0 |
| Arctiini | *Virbia sanguicollis* | 12.5 | 1 | 0 | 0 |
| Arctiini | *Viviennea salma* | 15.4 | 1 | 0 | 0 |
| Arctiini | *Viviennea tegyra* | 19.8 | 1 | 0 | 0 |
| Arctiini | *Watsonidia reimona* | 14.2 | 1 | 0 | 0 |
| Arctiini | *Xanthyda saron* | 13.2 | 1 | 1 | 0 |
| Lithosiini | *Abrochocis esperanza* | 4.9 | 1 | 0 | 0 |
| Lithosiini | *Agylla* sp01 nr. *marginata* | 10.7 | 0 | 0 | 0 |
| Lithosiini | *Agylla* sp02 | 10.8 | 0 | 0 | 0 |
| Lithosiini | *Anaulosia* sp. | 6.5 | 0 | 0 | 0 |
| Lithosiini | *Balbura dorsisigna* | 13.2 | 1 | 0 | 0 |
| Lithosiini | *Cloesia digna* | 13.2 | 1 | 0 | 0 |
| Lithosiini | *Dolichesia falsimonia* | 6.9 | 1 | 0 | 0 |
| Lithosiini | *Epeiromulona* nr. *lephina* | 6.2 | 1 | 0 | 0 |
| Lithosiini | *Euthyone simplex* | 7.1 | 0 | 0 | 0 |
| Lithosiini | *Euthyone* sp01 | 5.8 | 0 | 0 | 0 |
| Lithosiini | *Euthyone* sp02 nr. *grisescens* | 10.0 | 0 | 0 | 0 |
| Lithosiini | *Illice citrina* | 6.1 | 1 | 0 | 0 |
| Lithosiini | *Illice croesus* | 6.1 | 1 | 0 | 0 |
| Lithosiini | *Illice* sp01 | 6.1 | 0 | 0 | 0 |
| Lithosiini | *Illice* sp02 | 6.1 | 0 | 0 | 0 |
| Lithosiini | *Illice tesselata* | 5.9 | 1 | 0 | 0 |
| Lithosiini | *Lycomorphodes sordida* | 8.5 | 1 | 0 | 1 |
| Lithosiini | *Macroptila* sp. nr. *rotundata* | 13.8 | 0 | 0 | 0 |
| Lithosiini | *Metalobosia* sp. | 6.9 | 0 | 0 | 0 |
| Lithosiini | *Nodozana hieroglyphica* | 5.4 | 1 | 0 | 0 |
| Lithosiini | *Nudur fractivittarum* | 5.8 | 1 | 0 | 0 |
| Lithosiini | *Odozana* nr. *methaemata* | 6.5 | 1 | 0 | 0 |
| Lithosiini | *Odozana* nr. *sixola* | 7.2 | 1 | 0 | 0 |
| Lithosiini | *Odozana* sp01 | 7.0 | 0 | 0 | 0 |
| Lithosiini | *Odozana* sp02 | 7.2 | 1 | 0 | 0 |
| Lithosiini | *Palaeozana mida* | 6.6 | 1 | 0 | 0 |
| Lithosiini | *Prepiella* sp. | 4.6 | 1 | 0 | 0 |
| Lithosiini | *Talara* sp01 nr. *minynthadia* | 6.6 | 1 | 0 | 0 |
| Lithosiini | *Talara* sp02 | 6.3 | 1 | 0 | 0 |
| Lithosiini | *Talara* nr. *mona* | 5.2 | 1 | 0 | 0 |
| Lithosiini | *Talara* nr. *rubida* | 6.0 | 1 | 0 | 1 |
