# Supplementary material for: Trait Variation in Moths Mirrors Small-Scaled Ecological Gradients in A Tropical Forest Landscape
Source: Insects. 2020 Sep 8;11(9):612. doi: 10.3390/insects11090612 (PMC7563231; doi:10.3390/insects11090612)
Supplement: Supplementary file 1 [file insects-11-00612-s001.zip › Table S2.docx]

**Table S2:** Fore-wing length of 258 species of moths in the family Geometridae, sampled with light-traps along two environmental gradients in the region around La Gamba (SW Costa Rica).

| **Species name** | **Fore-wing length (mm)** |
| --- | --- |
| *Aplogompha* nr. *costimaculata* | 11.9 |
| *Argyrotome* nr. *melae* | 14.4 |
| *Ballantiophora* nr. *gibbiferata* | 12.7 |
| *Betulodes matharma* | 26.7 |
| *Bryoptera* sp. | 16.5 |
| *Bryoptera subbrunnea* | 15.5 |
| *Chloropteryx* nr. *dealbata* | 10.6 |
| *Chloropteryx* nr. *opalaria* | 8.9 |
| *Chloropteryx* nr. *punctilinea* | 8.2 |
| *Chloropteryx* sp01 | 9.7 |
| *Chloropteryx* sp02 | 9.7 |
| *Chloropteryx* sp03 | 6.2 |
| *Cimicodes albicosta* | 21.2 |
| *Cyclomia minuta* | 5.2 |
| *Cyclomia vinosa* | 7.6 |
| *Cyclophora anablemma* | 5.9 |
| *Cyclophora melitia* | 14.0 |
| *Cyclophora nivestrota* | 15.7 |
| *Cyclophora nodigera* | 14.6 |
| *Cyclophora* nr. *griseomixta* | 15.7 |
| *Cyclophora* nr. *insiginata* | 18.3 |
| *Cyclophora* nr. *proconcava* | 8.1 |
| *Cyclophora* nr. *torsivena* | 8.2 |
| *Cyclophora ruficosta* | 12.1 |
| *Cyclophora* sp01 | 11.2 |
| *Cyclophora* sp02 | 13.7 |
| *Cyclophora* sp03 | 9.7 |
| *Cyclophora* sp04 | 11.7 |
| *Cyclophora* sp05 | 12.1 |
| *Cyclophora* sp06 | 10.3 |
| *Cyclophora* sp07 | 10.5 |
| *Dichorda obliquata* | 9.1 |
| *Dithecodes* sp01 | 7.4 |
| *Dithecodes* sp02 | 5.9 |
| *Dolichoneura* nr. *cinerea* | 14.8 |
| *Dolichoneura oxypteraria* | 14.1 |
| *Dolichoneura* sp. | 16.1 |
| *Dolichoneura squalida* | 15.6 |

| *Dyspteris* nr*. parvula* | 9.9 |
| --- | --- |
| *Dyspteris* nr. *tenuivitta* | 18.3 |
| *Dyspteris* nr. *vecinaria* | 14.4 |
| *Dyspteris* sp. | 9.7 |
| Ennominae indet. | 8.7 |
| *Eois apyraria* | 6.9 |
| *Eois coloraria* | 8.4 |
| *Eois* nr. *adimaria* | 8.4 |
| *Eois* nr. *antiopata* | 7.4 |
| *Eois* nr*. apyraria* | 7.0 |
| *Eois* nr. *guapa* | 7.0 |
| *Eois* nr. *particolor* | 8.1 |
| *Eois* nr. *plana* | 9.4 |
| *Eois* nr. *russearia* | 7.0 |
| *Eois* nr. *subtectata* | 7.5 |
| *Eois* nr. *tegularia*_01 | 7.0 |
| *Eois* nr. *tegularia*_02 | 6.3 |
| *Eois* nr. *undulosata* | 11.5 |
| *Eois* sp01 | 8.3 |
| *Eois* sp02 | 8.0 |
| *Eois* sp03 | 9.0 |
| *Eois zenobia* | 9.4 |
| *Epimecis* nr. *patronaria* | 28.2 |
| *Epimecis* nr. *subroraria* | 33.5 |
| *Epimecis patronaria* | 36.2 |
| *Erastria decrepitaria* | 17.8 |
| *Ergavia* nr. *carinenta* | 27.4 |
| *Ergavia* sp. | 23.5 |
| *Eubaphe* sp. | 12.8 |
| *Euclysia dentifasciata* | 21.4 |
| *Eumacrodes* nr. *certis* | 8.9 |
| *Eumacrodes* sp. | 6.7 |
| *Euphyia* sp01 | 16.4 |
| *Euphyia* sp02 | 15.9 |
| *Eupithecia* sp. | 5.1 |
| *Eusarca cayennaria* | 13.8 |
| *Eusarca crameraria* | 15.0 |
| *Eusarca flexilis* | 12.8 |
| *Eusarca minucia* | 14.4 |
| *Eusarca* sp01 | 11.8 |
| *Eusarca* sp02 | 13.8 |
| *Eutomopepla artena* | 15.3 |
| *Eutomopepla discuneata* | 18.5 |
| *Glena* nr. *subannulata* | 17.9 |
| *Hemipterodes* nr. *subrotundata* | 8.0 |
| *Hemipterodes subnigrata* | 8.2 |
| *Herbita lilacina* | 21.1 |
| *Herbita praeditaria* | 20.9 |
| *Hydata povera* | 7.4 |
| *Hydata* sp. | 7.8 |
| *Hymenomima* nr. *camerata* | 10.5 |
| *Hymenomima seriata* | 9.5 |
| *Hymenomima umbelularia* | 10.4 |
| *Hypometalla* sp. | 5.7 |
| *Idaea* nr. *subfervens* | 8.9 |
| *Idaea* sp01 | 9.9 |
| *Idaea* sp02 | 4.9 |
| *Idaea* sp03 | 4.0 |
| *Idaea* sp04 | 4.4 |
| *Idaea* sp05 | 5.8 |
| *Iridopsis oberthuri* | 19.6 |
| *Iridopsis pandrosos* | 18.3 |
| *Ischnopteris bifinita* | 22.3 |
| *Ischnopteris* nr*. illineata* | 21.9 |
| *Isochromodes epioneata* | 12.1 |
| *Isochromodes* sp01 | 8.4 |
| *Isochromodes* sp02 | 8.5 |
| *Isochromodes* sp03 | 11.2 |
| *Isochromodes* sp04 | 17.4 |
| *Leptostales delila* | 7.8 |
| *Leptostales* nr. *angulata* | 9.0 |
| *Leptostales* sp. | 7.8 |
| *Leptostales terminata nursica* | 7.1 |
| *Leuciris fimbriaria* | 8.9 |
| *Leuciris institata* | 6.5 |
| *Leucula festiva* | 21.8 |
| *Lissochlora manostigma* | 13.3 |
| *Lissochlora* sp. | 7.6 |
| *Lobocleta* nr*. malvina* | 3.8 |
| *Lobocleta subcincta* | 8.6 |
| *Macaria achetata* | 15.0 |
| *Macaria approximaria* | 17.3 |
| *Macaria bejucoaria* | 13.4 |
| *Macaria catualda* | 12.6 |
| *Macaria infusata* | 13.6 |
| *Macaria* nr. *gambarina* | 15.3 |
| *Macaria* sp01 | 17.3 |
| *Macaria* sp02 | 14.5 |
| *Macaria* sp03 | 14.7 |
| *Macaria* sp04 | 14.1 |
| *Melinodes* sp. | 8.9 |
| *Microgonia* nr. *rufaria* | 25.1 |

| *Nematocampa arenosa* | 8.2 |
| --- | --- |
| *Nematocampa completa* | 11.9 |
| *Nematocampa straminea* | 5.8 |
| *Nemoria adjunctaria* | 11.2 |
| *Nemoria* nr. *adjunctaria* | 10.6 |
| *Nemoria* nr. *defectiva* | 12.7 |
| *Nemoria* nr. *pacificaria* | 14.0 |
| *Nemoria* nr. *punctilinea* | 16.9 |
| *Nemoria* nr. *scriptaria* | 13.9 |
| *Nemoria* sp. | 11.9 |
| *Neothysanis imella* | 6.8 |
| *Nepheloleuca politia* | 20.6 |
| *Oospila concinna* | 13.4 |
| *Oospila* nr. *albipunctulata* | 7.8 |
| *Oospila* nr. *atopochlora* | 18.7 |
| *Oospila* nr. *rubescens* | 17.2 |
| *Oospila* sp. | 22.7 |
| *Opisthoxia bella* | 15.4 |
| *Opisthoxia* nr. *bolivari* | 12.3 |
| *Opisthoxia* nr. *formosante* | 11.9 |
| *Opisthoxia* nr. *molpadia* | 10.0 |
| *Opisthoxia* sp01 | 12.5 |
| *Opisthoxia* sp02 | 10.8 |
| *Oxydia apidania* | 26.8 |
| *Oxydia* sp. | 30.7 |
| *Pachycopsis tridentata* | 6.6 |
| *Palyas micacearia* | 20.3 |
| *Paragonia cruraria* | 25.3 |
| *Paragonia* nr. *tasima* | 23.3 |
| *Parilexia cermala* | 11.5 |
| *Patalene aenetusaria* | 15.2 |
| *Patalene falcularia* | 12.1 |
| *Patalene luciata* | 16.8 |
| *Patalene* sp01 | 17.5 |
| *Patalene* sp02 | 13.8 |
| *Perigramma marginata* | 20.9 |
| *Perigramma* nr. *albivena* | 15.4 |
| *Perigramma* nr. *celerenaria* | 21.1 |
| *Perigramma* nr. *marginata* | 19.1 |
| *Perigramma* sp. | 21.5 |
| *Perissopteryx* nr. *gamezi* | 15.1 |
| *Perissopteryx* nr. *submarginata* | 17.1 |
| *Pero chapela* | 21.4 |
| *Pero simila* | 20.8 |
| *Pero* sp. | 22.2 |

| *Phrudocentra albicoronata sixola* | 13.0 |
| --- | --- |
| *Phrudocentra janeira tenuis* | 14.4 |
| *Phrudocentra neis neis* | 14.1 |
| *Phrudocentra pupillata* | 11.9 |
| *Phrygionis privignaria* | 15.0 |
| *Phyllodonta* sp01 | 17.4 |
| *Phyllodonta* sp02 | 14.6 |
| *Physocleora* sp01 | 13.9 |
| *Physocleora* sp02 | 15.5 |
| *Physocleora* sp03 | 17.7 |
| *Physocleora* sp04 | 6.2 |
| *Plemyriopsis* nr*. facetata* | 7.4 |
| *Pleuroprucha* sp01 | 8.8 |
| *Pleuroprucha* sp02 | 7.8 |
| *Pleuroprucha* sp03 | 8.4 |
| *Polla hemeraria* | 18.6 |
| *Procherodes tetragonata* | 24.8 |
| *Proutoscia mirifica* | 18.9 |
| *Ptychamalia* nr. *simplex* | 8.1 |
| *Pyrinia helvaria* | 13.6 |
| *Pyrinia* nr. *augustata* | 10.5 |
| *Pyrinia* sp01 | 14.5 |
| *Pyrinia* sp02 | 10.0 |
| *Pyrinia* sp03 | 11.7 |
| *Pyrochlora rhanis* | 11.5 |
| *Rhodochlora* nr. *brunneipalpis* | 18.9 |
| *Sabulodes carbina* | 13.8 |
| *Scopula compensata* | 7.3 |
| *Scopula* sp. | 6.6 |
| *Scopula umbilicata* | 8.0 |
| *Semaeopus caparonensis* | 10.7 |
| *Semaeopus exquisata* | 14.7 |
| *Semaeopus miniata* | 10.3 |
| *Semaeopus* nr. *fulvescens* | 11.6 |
| *Semaeopus* nr. *illimiata* | 15.7 |
| *Semaeopus* nr. *masinissa* | 11.0 |
| *Semaeopus* nr. *miniata* | 10.4 |
| *Semaeopus* nr. *peplumaria* | 11.8 |
| *Semaeopus* nr*. semibrunnea* | 10.9 |
| *Semaeopus* nr. *tropaea* | 13.8 |
| *Semaeopus peplumaria* | 9.2 |
| *Semaeopus* sp. | 12.7 |
| *Semiothisa salsa* | 15.2 |
| *Semiothisa* sp. | 8.9 |
| *Sericoptera reductata* | 19.8 |
| *Spargania* sp. | 13.6 |

| *Sphacelodes quadrilineata* | 17.8 |
| --- | --- |
| *Sphacelodes vulneraria* | 20.2 |
| *Synchlora expulsata* | 6.5 |
| *Synchlora gerularia* | 7.7 |
| *Synchlora* nr. *concinnaria* | 6.7 |
| *Synchlora* nr. *pomposa* | 9.8 |
| *Synchlora pulchrifimbria* | 5.9 |
| *Synchlora superaddita* | 6.9 |
| *Tachyphyle albisparsa* | 15.7 |
| *Tachyphyle oleaster* | 12.7 |
| *Telotheta* sp. | 13.1 |
| *Tetragonodes anopsaria* | 14.0 |
| *Thyrinteina arnobia* | 18.0 |
| *Thysanopyga abdominaria* | 17.2 |
| *Thysanopyga nigristicta* | 12.6 |
| *Thysanopyga* nr. *amarantha* | 16.6 |
| *Thysanopyga* nr. *gauldi* | 18.0 |
| *Thysanopyga* nr. *pygaria* | 15.4 |
| *Thysanopyga* sp. | 15.7 |
| *Tmetomorpha bitias* | 16.7 |
| *Tricentra gavisata* | 6.3 |
| *Tricentra quadrigata* | 6.4 |
| *Tricentra* sp01 | 6.4 |
| *Tricentra* sp02 | 6.5 |
| *Tricentra* sp03 | 7.1 |
| *Tricentra* sp04 | 6.6 |
| *Tricentra* sp05 | 5.7 |
| *Tricentra* sp06 | 6.1 |
| *Tricentra* sp07 | 6.7 |
| *Tricentra unimacula* | 8.2 |
| *Tricentrogyna* sp01 | 5.1 |
| *Tricentrogyna* sp02 | 5.1 |
| *Tricentrogyna* sp03 | 3.8 |
| *Tricentrogyna* sp04 | 6.3 |
| *Tricentrogyna* sp05 | 20.6 |
| *Tricentrogyna* sp06 | 3.8 |
| *Zanclopteryx* sp. | 7.1 |
